# Sensing of Salivary Glucose Using Nano-Structured Biosensors

**DOI:** 10.3390/bios6010010

**Published:** 2016-03-17

**Authors:** Yunqing Du, Wenjun Zhang, Ming L. Wang

**Affiliations:** 1 Interdisciplinary Engineering Program, College of Engineering, Northeastern University, 360 Huntington Ave, 400 SN, Boston, MA 02115, USA; zhang.wenj@husky.neu.edu (W.Z.); mi.wang@neu.edu (M.L.W.); 2 Civil and Environmental Engineering, and Bioengineering Department, Northeastern University, 360 Huntington Ave, 429 SN, Boston, MA 02115, USA

**Keywords:** saliva diagnostics, noninvasive, diabetes, glucose monitoring, biosensor, layer-by-layer assembly

## Abstract

The anxiety and pain associated with frequent finger pricking has always been troublesome for diabetics measuring blood glucose (BG) in their daily lives. For this reason, a reliable glucose monitoring system that allows noninvasive measurements is highly desirable. Our main objective is to develop a biosensor that can detect low-level glucose in saliva (physiological range 0.5–20 mg/dL). Salivary glucose (SG) sensors were built using a layer-by-layer self-assembly of single-walled carbon nanotubes, chitosan, gold nanoparticles, and glucose oxidase onto a screen-printed platinum electrode. An electrochemical method was utilized for the quantitative detection of glucose in both buffer solution and saliva samples. A standard spectrophotometric technique was used as a reference method to validate the glucose content of each sample. The disposable glucose sensors have a detection limit of 0.41 mg/dL, a sensitivity of 0.24 μA·s·dL·mg^−1^, a linear range of 0.5–20 mg/dL in buffer solution, and a response time of 30 s. A study of 10 healthy subjects was conducted, and SG levels between 1.1 to 10.1 mg/dL were successfully detected. The results revealed that the noninvasive SG monitoring could be an alternative for diabetes self-management at home. This paper is not intended to replace regular BG tests, but to study SG itself as an indicator for the quality of diabetes care. It can potentially help patients control and monitor their health conditions, enabling them to comply with prescribed treatments for diabetes.

## 1. Introduction

Diabetes mellitus is considered to be the most rapidly growing chronic disease of this century. It is a disease in which a person suffers from high blood sugar, either because the pancreas does not produce enough insulin (Type I), or because cells do not respond to the insulin that is produced (Type II). In 2014, 29.1 million Americans, about 9.3% of the total US population, were diagnosed with diabetes [1]. Besides inducing negative impacts on the life quality of patients, the epidemic of diabetes is resulting in more costs to the government health care budget each year. For the self-management of diabetes, most patients need to test their blood glucose (BG) levels periodically, actively dosing with insulin, or taking an oral drug in combination with controlling their diet. Typically, a blood sample for analysis is obtained through a finger prick, which often causes physical and mental stress to patients. Therefore, there is a great need for a point-of-care glucose monitoring system that allows for noninvasive, rapid and painless measurements. Saliva has been studied as a better indicator of disorders and diseases than blood [2]. Specifically, the salivary glucose (SG) level is considered an indicator of diabetes. According to preliminary studies, a good correlation between the BG level and the SG level has been verified [3,4,5,6]. Thus, the tracking of BG through the continuous monitoring of SG levels is very promising. Saliva offers great advantages as a diagnostic fluid over other body fluids such as blood, tears, sweat, urine and so forth. First, it is easily tested by individuals with modest training; second, it is noninvasive, so the risk of infection or cross-contamination caused by frequent finger pricks is eliminated; third, it is convenient for people who face difficulty extracting blood samples such as infants, the elderly and haemophiliacs; and, last but not the least, saliva contains numerous disease-related biomarkers, including those typically found in blood [7]. Taking these into account, this study developed a noninvasive SG sensing system which can be transformed into a saliva-based multiplex biomarkers detection system for the management of diabetes.

Various technologies, including infrared spectroscopy, fluorescence spectroscopy, raman spectroscopy, liquid chromatography–mass spectrometry (LC-MS) and gas chromatography–mass spectrometry (GC-MS), were proved capable of detecting glucose in clinical specimens [8,9,10,11,12]. However, the requirements of expensive equipment and complicated operation prohibited them from being applied to home-based care. Hence, SG sensors introduced in this study were developed based on electrochemical sensing technology, which has attracted more and more attention due to its high selectivity, repeatability, accuracy, strong biocompatibility, and low cost [13]. A typical sensor consists of a bioreceptor and transducers. The bioreceptor recognizes a target analyte and specifically reacts with it. Transducers are then used to convert the recognition event into a measurable electrical signal. For the purpose of glucose detection, the most commonly used bioreceptor is glucose oxidase (GOx) enzyme. In 1978 the first BG biosensor was designed relying on catalytic enzyme reactions [14]. It detected the hydrogen peroxide (H_2_O_2_) product that was generated from the glucose and GOx reaction when oxygen (O_2_) was present. However, the measurement of H_2_O_2_ was easily altered by the product (H_2_O_2_) accumulation and also limited by the concentration of O_2_ [15]. Due to these concerns, the second-generation glucose sensor was rendered by replacing O_2_ with a synthetic electron acceptor (mediator), which was capable of shuttling electrons from the redox center of the enzyme to the surface of the electrode [13]. Measuring the electron transfer carried by mediators instead of products has successfully eliminated the drawbacks of the first generation. Nevertheless, there were still unsolved problems such as the potential leaching, low stability, and toxicity of mediators. That was when the third-generation glucose sensor came along, with the promise of a mediator-free glucose detection system based on direct electron transfer (DET). It is crucial to have a short distance or efficient electron-transfer pathway between the electrode and the reaction site which allows DET to happen. The third generation eliminated the interferences caused by O_2_ concentration, H_2_O_2_ accumulation, and mediators leaching during the reaction, so more stable and precise electron transfer was captured [16]. Because of these advantages, we developed a third-generation glucose sensor using screen-printed sensor chips as bases.

Screen printing technology was widely used to mass produce disposable sensor chips. This technology was favored since it had flexible choices of cheap bulk materials, a mature process development, a fast production line, and high manufacturing capability and capacity. Table 1 displays a list of screen-printed glucose biosensors: here, it is evident that the third-generation glucose sensors achieved a lower limit of detection (LOD) with a faster response time compared to the first- and second-generation. Some sensors in this table display a broader range of detection or a faster response time when compared to our sensors. However, none of them have ever successfully detected glucose from real saliva samples. Unlike the other similar electrochemical biosensors reported in literature, developed sensors in this study demonstrate the capability of a real time SG measurement within 2 min (including saliva collection and testing). In addition, sensors presented in this paper provide a sufficient glucose detection range of 1.1–45 mg/dL, pinpointing the biological range of human SG (0.5–20 mg/dL), permitting them to be applicable for diabetic control and health surveillance [17]. Extremely low SG levels (<1.1 mg/dL) and high SG levels (>10.1 mg/dL) have not been examined yet since saliva samples of diabetic patients are excluded from this study. Here, an accurate, rapid, and portable SG measurement method is introduced to improve its clinical practicability and reduce pain for real-time diabetes management. Moving forward, our future goal is to measure extremely low/high SG levels in patients with diabetes as part of SG clinical validation study.

## 2. Experimental Section

In this study, the developed glucose-sensing system can accurately measure the SG levels of healthy subjects. Sensors were fabricated through a layer-by-layer (LBL) self-assembly process on screen-printed electrodes. They were designed to help diabetics manage their health conditions and treatment results by monitoring SG levels. However, this study is not intended to replace BG tests. We believe that SG itself could potentially be considered as means for diabetes monitoring. The clinical applicability of SG sensors will be clearer especially when the next steps of clinical studies are finished.

### 2.1. Sensor Fabrication

**Figure 1:**
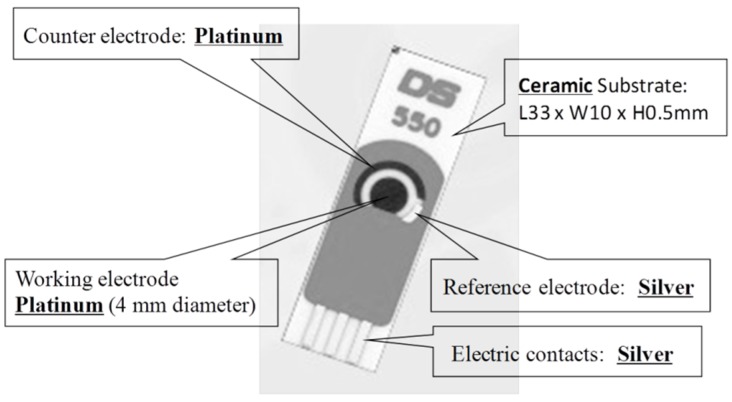
Schematic of screen-printed three-electrode sensor chips.

DS550 platinum (Pt) screen-printed sensor chips were purchased from DropSens (Metrohm USA Incorporated). The three metallic electrodes were a Pt working electrode (WE), a silver (Ag) reference electrode (RE), and a Pt counter electrode (CE) (Figure 1). Phosphate buffered saline (PBS, pH 7.4, Sigma-Aldrich Co. LLC.) was dissolved in deionized (DI) water to yield 0.1 M PBS aqueous buffer solution. Carboxyl groups functionalized single-walled carbon nanotubes (SWNT–COOH, diameter: 1~2 nm; length: 2~5 μm, 4000 mg/L in DI water with 5~7 wt% COOH groups at the ends) were obtained from Brewer Science Company. Chitosan (CS, low molecular weight), spherical gold nanoparticles (GNp, colloid gold, 20 nm diameter stabilized suspension), GOx (type II lyophilized powder with at least 17,300 units/g solid, enzyme commission (EC) 1.1.3.4 enzyme from Aspergillus niger), and acetate buffer solution (pH 4.65) were purchased from Sigma-Aldrich Co. LLC. Before use, 400 μL of the SWNT suspension was dispersed in 10 mL DI water with the aid of ultrasonication for 2 h to obtain stable black stock suspension; 10 mg CS was dissolved thoroughly into 5 mL acetate buffer solution to yield 2 mg/mL CS suspension; 5 mg GOx was dissolved into 5 mL 0.1 M PBS to produce 1 mg/mL GOx suspension. Experiments were performed at room temperature at approximately 23 °C.

For sensor fabrication, all sensors were first rinsed with DI water and left to air dry. Only working electrodes were exposed while the other two electrodes were covered with dielectric tape (MSC industrial supply Co.). Then, 10 μL of SWNT suspension was cast onto each sensor and allowed to dry in desiccator (Terra Universal) under 10% relative humidity (RH). After 25 min, sensors were washed with 0.1 M PBS and dried up. The washing step was applied after the deposition of each layer (unless otherwise stated). Then, 10 μL of 2 mg/mL CS, 10 μL of GNp, and 10 μL of 1mg/mL GOx were cast onto the exposed electrode sequentially to form the first (CS/GNp/GOx) multi-layer film: each layer took 20 min to complete. Subsequently, two more multi-layer films were cast, and sensors were dried in the desiccator for 1 h without a washing step. After removing the dielectric tape, all resulting sensors were packed in gel-boxes (Gel-Pak) and then sealed in vacuum bags using a vacuum packaging machine (VACmaster pro110). Sensors were stored at 4 °C when not in use.

### 2.2. Micro-Fabrication Imaging

All micro-fabrication images were produced using a Supra 25 scanning electron microscope (SEM) from Gorge J. Kostas Nanoscale Technology and Manufacturing Research Center located at Northeastern University.

### 2.3. Sensor Measurement

Electrochemical measurements were carried out using a potentiostat (DY2100 mini, Digi-ivy), which was connected to a laptop with pre-installed post data processing software. A boxed adaptor for solid connection between single-use three-electrode sensors and potentiostat was purchased from DropSens. First, cyclic voltammetry (CV) electro-analytical tests were conducted and a suitable voltage to be applied between the WE and the RE was determined, under which the output current between the WE and the CE corresponded to the change of glucose concentrations. During the test, the applied voltage was sweeping from −0.2 V to 0.4 V at a scan rate of 50 mV/s. Current and voltage profiles of the third cycle were analyzed to determine a proper working potential. Then under a fixed working potential, amperometric tests were conducted to measure the glucose level, which is proportional to the output current signal. A 100 μL sample was dropped to cover all three electrodes before the selected voltage was applied between the WE and the RE constantly for 30 s. As glucose-GOx redox reactions happened on the WE, the output current between the WE and the CE illustrated how much charge passed along, quantitatively indicating the amount of glucose. In short, glucose concentrations were determined as a function of output current densities. As the reactions reached a steady state within 30 s, the output current in a time window of 27~30 s was integrated and used as the analytical signal. At the end of this 30 s test, a matlab script ran automatically to analyze the data and display results on a laptop.

### 2.4. Glucose Analysis with Reference Method

Spectrophotometric analysis based on enzymatic reactions by using ultraviolet-visible (UV-vis) spectroscopy was considered as a standard reference method for quantitative detection of glucose. Glucose assay kits (K606-100, Biovision incorporated) were used along with an ultraviolet (UV) spectrophotometer (mini1240, Shimadzu) and ultra-micro UV cuvettes (Brandtech scientific incorporated). This method detects 0.018–180 mg/dL glucose samples with a resolution of 7.2 × 10^−5^ mg/dL [29]. For each batch of measurements, a set of six standard glucose solutions were prepared following the manufacturer’s protocol to provide a calibration curve. Samples were incubated in a water bath at 37 °C for 30–40 min and tested at a 570 nm wavelength. Absorbance readings were then converted into glucose concentrations using the calibration curve. All glucose concentrations in saliva/buffer samples were validated with standard UV tests.

### 2.5. Blood Glucose and Salivary Glucose Monitoring Test

Ten healthy volunteers in an age group of 20–60 years participated in an anonymous human subjects study. Diabetic and pre-diabetic subjects were excluded from this study. This project protocol was approved by Institutional Review Board of Northeastern University Human Subject Research Protection in February 2013 and was identified as #12-11-31. Each subject signed consent forms after fully understanding the purpose, procedure, and risks of the study, and were offered $12 compensation to purchase a lunch box at the completion of each session. Subjects were required to fast overnight without drinking/eating anything (except water) after 10 p.m. prior to the test date. In the morning, both blood and saliva samples of each subject in their fasted state were taken for analysis. Blood samples were measured by finger prick method using FreeStyle Lite BG monitoring systems (Abbott). Saliva samples for sensor tests were collected within 1–2 min following the saliva collection protocol as described in Section 2.6. The pH and viscosity of saliva samples were also recorded using pH test papers (6.0–8.0 range, FisherBrand) and a portable viscometer (Core-Parmer). The remaining saliva samples were prepared for spectrophotometric analysis. The preparation procedures included boiling samples at 100 °C for 30 to 60 min, and centrifuging them at 12,000× g for 6 min. The supernatant was then collected and analyzed for glucose concentrations as a reference method.

### 2.6. Saliva Collection Protocol

(a) Wait for 5 min after rinsing mouth with water;
(b) Minimize swallowing and hold saliva in mouth;
(c) Place sterilized dental cotton sponge in mouth and chew until it is soaked with saliva (typically < 1 min);
(d) Deposit sponge into syringe directly from the mouth without touching it to avoid contamination;
(e) Insert plunger into syringe;
(f) Squeeze saliva through pre-installed Westran S 0.2 μm polyvinylidene fluoride (PVDF) membrane (Sigma-Aldrich Co. LLC.) at the bottom of syringe and into sterilized tubes. Usually 1 ml of saliva samples is obtained through this process;
(g) Use pipette to drop 100 μL saliva onto a sensor to cover all three electrodes;
(h) Record measurement;
(i) Dispose of sensor after washing out residual salivary specimen.

The saliva collection method provides a reliable filtering performance to remove large biomolecules such as mucins from saliva samples. The collected saliva’s viscosity usually ranges from 1.05 to 1.15 mPa·s, which is close to the viscosity of buffer solutions [30]. This is because the 0.2 μm pore size filter that comes pre-installed in collection devices is comprised of a fine PVDF material, which is widely used for protein blotting due to its high protein binding capability (over 200 µg/cm^2^) [31]. Its high efficiency in mucins removal eliminates the matrix effects for saliva tests.

## 3. Results and Discussion

### 3.1. Layer-by-Layer Bio-Layer Construction

A variety of methods including physical adsorption, cross-linking, sol-gel matrix entrapment, covalent binding, membrane entrapment, and LBL self-assembly are widely used to fabricate glucose sensors. Among them, LBL self-assembly offers better uniformity, stability, sensitivity; it is also cost effective and has an easier operation processes [32]. This is due to the following: first, there are multiple interaction forces such as electrostatics forces, hydrogen bonds, covalent bonds, Van der Waals’ interactions and hydrophobic interactions that we can choose from to build the most appropriate layer composition [33]; second, the LBL films often demonstrate close identical properties after deposition, no matter what substrate they are sitting on [34]; and, third, there is no complicated operation process involved, other than simply dipping the WE into different coating reagents. Thus, the LBL self-assembly method was selected for sensor fabrication.

Distinguished from biosensors that are fabricated for BG testing, SG sensors are developed using nano-materials to achieve better sensitivity, a lower detection limit, and a sufficient detection range for SG measurements. The bioreceptor GOx was used as a catalyst in the oxidation of glucose. During this reaction, electrons were transferred from the coenzyme flavin adenine dinucleotie (FAD) moieties to electrodes through the assembled transducers. Three transducers, SWNT-COOH, CS, and GNp, were selected and optimized in composition. Recent studies demonstrated that carbon nanotubes (CNT) promoted electron transfer reactions and enhanced the electrochemical reactivity of enzymes such as GOx [35]. SWNT was selected over multi-walled CNT due to its higher field emission current, conductivity and specific surface area [36]. The hydrophobic nature of SWNT also permitted it to easily bind onto Pt electrodes through a hydrophobic attraction. With its highly specific surface area and molecule capture capability, SWNT was well confined as a base layer. Wang’s group [35] proved that CNT disturbed the secondary structure of GOx and forced its redox centers to move close to electrodes’ surface, causing electrons to pass directly without mediators. By applying SWNT, DET from the enzyme to the electrode was realized via a series of “wired” relay centers to achieve lower glucose detection limit. CS was selected due to its outstanding biocompatibility, nontoxicity, stability, and low cost. It contains large numbers of protonated amino groups (–NH^3+^) and hydroxyl groups (–OH) functional groups and is widely used as a bio-linker for enzyme immobilization. GNp has been commonly used in the design of electro-chemical sensors. As an electrocatalyst, GNp can promote electron transfer reactions and dramatically increase the specific surface area to capture a large amount of enzymes so that sensors’ sensitivity is enhanced accordingly [37].

**Figure 2:**
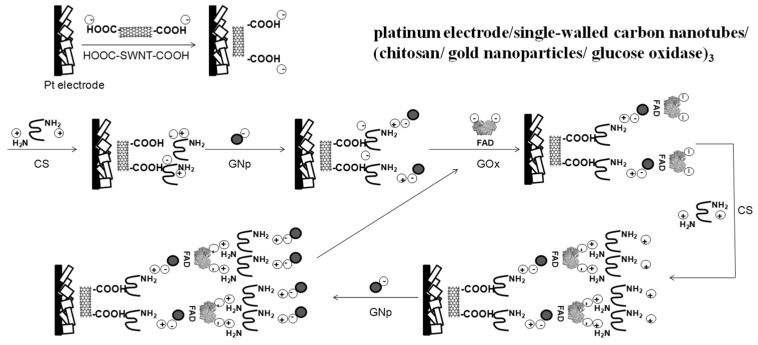
Schematic illustration of the layer-by-layer self-assembly procedure.

The stepwise modification process is described in Figure 2 with detailed information on composition layers [38,39,40]. The washing step between each layer was to remove un-bound chemicals. SWNT was first entrapped onto the porous Pt WE surface through physical adsorption, hydrophobic attraction, and electrostatic forces. As reported by the manufacturer, their carboxylation process yielded SWNT–COOH with a strong negative charge, while CS carried positive charges which were contributed by protonated amine groups. Thus, the first CS layer was deposited and linked to the SWNT film through electrostatics forces due to their opposite electric charges. Likewise, bonds between GNp and CS were built through electrostatics forces relying on their opposite electric charges. After this, GOx with negatively charged FAD moieties were assembled onto the GNp layer through physical and covalent adsorption [41]. Some un-adsorbed GOx was also entrapped into underlayer matrices through physical adsorption as well as electrostatics interactions. After fabricating the first (CS/GNp/GOx) multi-layer film, the number of films was optimized based on the sensors’ performance. The isoelectric point of GOx is reported as 4.2 by Sigma-Aldrich Co. LLC [42]. So under the enzyme immobilization process when the pH was 7.4, the overall net charge on GOx was negative. When the second multi-layer film was applied, GOx and CS bonds were formed through electrostatic interactions. The rest of the process was repeated as more layers were added.

**Figure 3:**
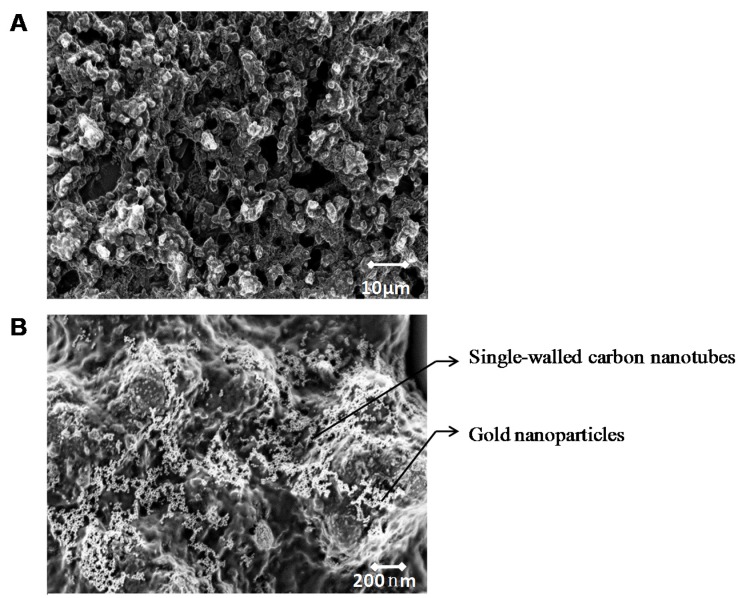
(**A**) Scanning electron microscope (SEM) image of platinum (Pt) electrode surface. (**B**) SEM image of one layer Pt/single-walled carbon nanotubes/chitosan/gold nanoparticles film on Pt electrode surface.

A surface imaging technique, SEM was used to obtain information about sensor surface topography and composition. The selected screen-printed sensor chips chose Pt as WE for two reasons: first, its coarse surface morphology with gaps and pinholes permits its large surface areas to entrap many biomolecules (Figure 3A); second, as a noble metal, Pt is chemically inert and tends to provide a stable working potential. Another noble metal, Ag, was chosen as RE because it did not participate in reactions and, as a result, prevented electrode fouling.

In the SEM image in Figure 3B, a single layer of GNp (white dots) was well dispersed on the SWNT (white short bars) formed matrix layer. GNp’s comparable size to GOx provided GOx with more active binding sites and, as a result, immobilized more enzymes through physical adsorption and electrostatics force [43]. Although the covalent attachment method is also widely used to immobilize GOx with high stability, it normally leads to moderate or severe inactivation of enzymes due to conformational changes induced by the strong bonds [43]. Therefore, higher enzyme reactivity is retained through this LBL method since the applied adsorption method does not change the secondary structure of GOx. Here, the SEM image illustrates that multi-layers films are well distributed and provided large surface areas for GOx immobilization.

### 3.2. Number of Bio-Layers

**Figure 4:**
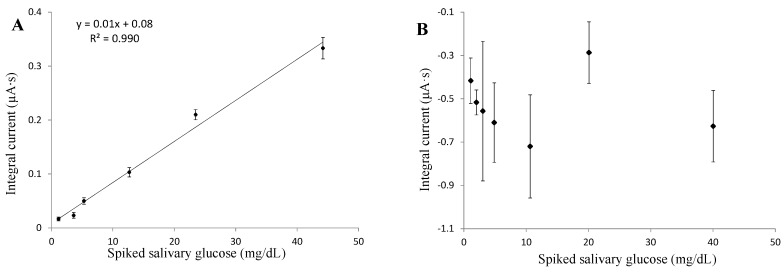
Amperometric tests with effect of different number of (chitosan/gold nanoparticles/glucose oxidase) multi-layer films on electrode (**A**) three multi-layer films; (**B**) six multi-layer films. Error bars = ±standard deviation and *n* = 3.

The working potential was determined to be as low as 0.05 V for three layers, and 0.2 V for six layers, through CV tests. Saliva samples were treated through filtration, boiling and centrifuging processes, and then spiked with glucose to attain different glucose concentrations. Glucose was spiked as concentrated solution by adding 1 part solution to 40 parts treated saliva. The saliva matrix effect was eliminated because the viscosity of spiked saliva samples was close to the viscosity of the buffer solution (about 1.1 mPa·s). By comparing the amperometric results of three and six layers of multi-layer film fabricated sensors, the three-layered film demonstrated a better sensing linearity, intra-repeatability, and lower detection limit (Figure 4A,B). Both tests demonstrated that the absolute values of current density were proportional to glucose concentrations under a fixed working potential. However, the direction of current flow was different with these two type of sensors. This is because, when conducting CV tests, the working potential was picked in a region where anodic current dominated (forward sweep) with three layers of sensors, while with six layers sensors it dropped in a region where cathodic current dominated (backward sweep). With a (CS/GNp/GOx)_3_ layer composition, sensors achieved a linear glucose detection range up to 45 mg/dL, and the linear regression was with 99% confidence limits. Theoretically, although films with more layers can immobilize larger amounts of GOx, films become thicker and so conductivity is reduced. Moreover, the extended distance between GOx and electrodes may eliminate DET and cause major interferences to glucose detection. As a result, three-layers composition was applied to achieve better sensor performance with less cost of materials and a faster fabrication process.

### 3.3. Glucose Sensing

**Figure 5:**
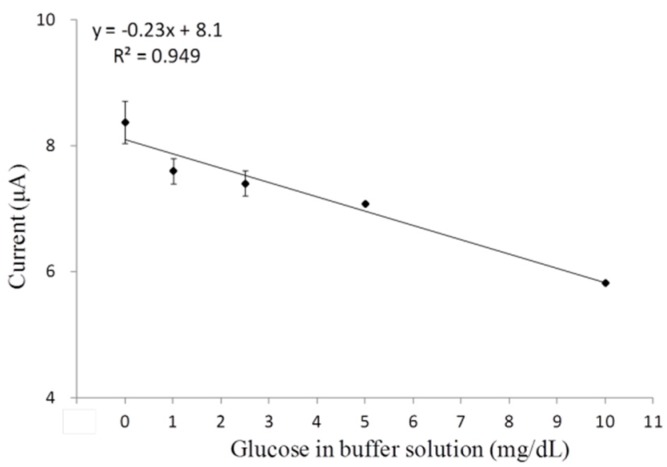
Cyclic voltammetry tests determined steady-state calibration curve of the single-walled carbon nanotubes/(chitosan/gold nanoparticles/glucose oxidase)_3_ functionalized platinum electrode. Error bars = ±standard deviation and *n* = 3.

With fixed three-layer composition, an appropriate working potential as low as 0.05 V was determined for amperometric tests. A high voltage may include many other redox reactions and cause interferences to glucose detection; thus, a consistent minimum voltage is required to capture all electron transfers for the glucose-GOx reactions alone. Through CV analysis, only the third cycles were analyzed because the signal tended to be more stable after two cycles (48 s). An extremely low working potential was selected with the use of elevated glucose concentrations in PBS, and the calibration curve at steady-state was plotted in Figure 5. This low applied potential further confirmed the DET between enzyme’s active sites and the electrode surface [44].

**Figure 6:**
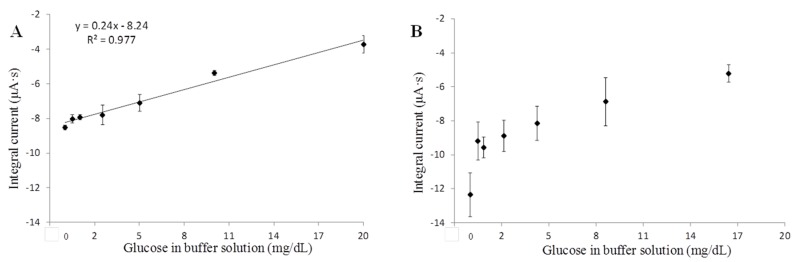
Amperometric tests with the effect of SWNT in support of sensor functionalization (**A**) single-walled carbon nanotubes/(chitosan/gold nanoparticles/glucose oxidase)_3_ functional layers; (**B**) (chitosan/gold nanoparticles/glucose oxidase)_3_ functional layers without single-walled carbon nanotubes. Error bars = ±standard deviation and *n* = 3.

Amperometric tests of Pt/SWNT/(CS/GNp/GOx)_3_ functionalized sensors were conducted under a selected working potential by using elevated glucose in PBS. It demonstrated a linear detection range of 0.5–20 mg/dL and the linear regression was with 98% confidence limits (Figure 6A). In order to study the SWNT’s effect on layer composition and sensing efficiency, the same tests with Pt/(CS/GNp/GOx)_3_ functionalized sensors were conducted under optimized working potential of −0.05 V. The results showed less repeatable readings with weaker linearity within this glucose range (Figure 6B). The comparison of sensor performances in Table 2 demonstrated sensors with SWNT had higher intra-repeatability, linearity, and lower LOD. Through this study, the high repeatability benefited from a uniformly confined SWNT matrix and well stabilized LBL multi-layer films on electrodes [45]. Concurrently, a lower glucose level was detectable in favor of the better conductivity and signal-to-noise ratio with the aid of SWNT [46]. The good linearity revealed that the immobilized GOx amount was sufficient for glucose detection up to 20 mg/dL, and that benefited from the large specific surface area provided by SWNT. As a result, this study concluded that SWNT played an essential role in promoting more repeatable measurements (coefficient of variance <10%), lower LOD (0.41 mg/dL), and a sufficient linear detection range (0.5–20 mg/dL with R^2^ = 0.98). With optimized sensor layer composition, a human subjects study was conducted to evaluate the sensors’ performance in SG measurements.

### 3.4. Glucose in Saliva

**Figure 7:**
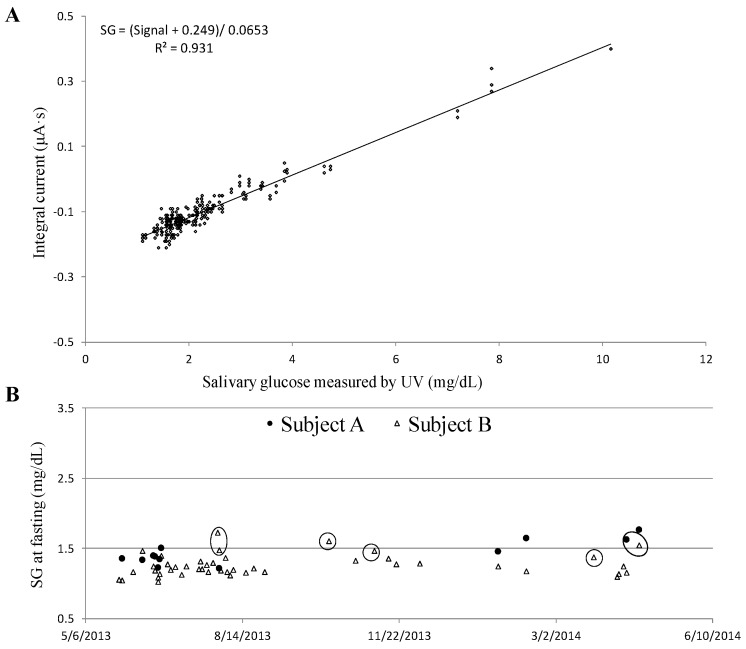
(**A**) Linear correlation between sensor and ultra-violet visible (UV) measured salivary glucose (SG) with obtained conversion formula; (**B**) Fasting SG monitoring history of two healthy females.

Two major objectives of the human subjects studies were as follows: (1) to understand the relationship between BG and SG of healthy people at a fasting state; (2) to screen for diabetes by measuring SG at fasting. Saliva samples were collected through the saliva collection protocol and tested immediately using glucose sensors. A linear correlation between the sensor’s signal and the reference method (UV method) that measured SG concentrations was found in a range of 1.1 to 10.1 mg/dL (R^2^ = 0.931), including 287 individual sensors’ measurement (Figure 7A). A conversion formula SG = (Signal + 0.249)/0.0653 mg/dL was derived from the linear regression curve (Figure 7A), and applied to convert sensors’ signal into SG levels. The intra-repeatability of SG levels measurements by at least three single-use glucose sensors achieved a variance <15%. Technically, BG levels at fasting are considered as a crucial index to evaluate peoples’ health conditions and, more importantly, as a tool to screen for diabetes. Normal BG levels are typically less than 100 mg/dL, while diabetes is diagnosed if the fasting BG is 126 mg/dL or higher [47,48]. In this study, subjects A and B demonstrated that their fasting BG levels were lower than 100 mg/dL. Correspondingly, their SG levels were monitored over a year and plotted in Figure 7B. The normal range of fasting SG was 1.4 to 1.6 mg/dL for subject A, and 1.1 to 1.4 mg/dL for subject B, respectively. There were a couple of data points (circled) when subjects were not in good health conditions, either having a cold/fever or were in menstruation. That was when an elevated SG level was recorded. In conclusion, the healthy individual fasting SG was relatively consistent over time as long as the subjects’ health conditions were unchanged.

Similar tests were further performed by eight healthy subjects. An interesting finding that was the BG (mg/dL)/SG (mg/dL) ratio of each subject at fasting was unique and the value was individually dependent (Table 3). For instance, subject B’s fasting BG/SG ratio was 69.8 with a variance of 7.1% based on a 39-day study; for subject A, the fasting BG/SG ratio was 64.8 with a variance of 10.8% based on an 11-day study. Thus, with the aid of an initial calibration to determine the individual’s BG/SG ratio at fasting, BG levels are predictable by measuring fasting SG levels on a daily basis. Thus, we propose an alternative method by measuring SG levels at fasting to evaluate glycemic levels and monitor health conditions. The findings indicate that SG levels at fasting can potentially be used to screen for diabetes. In the future, complete clinical validation including diabetic patients are crucial to apply the same concept obtained from this study for appropriate self-management of diabetes.

## 4. Conclusions

A disposable electrochemical sensor which could detect trace glucose levels down to 0.41 mg/dL has been developed. With a linear glucose detection range of 1.1–45 mg/dL, the sensors demonstrate good accuracy in SG measurements with the detection of variances of less than 15%. The human subjects studies, which included ten healthy subjects, have successfully accomplished a SG detection range of 1.1–10.1 mg/dL. Validation through the use of a reference UV method proves that the SG sensors are capable of accurate and reliable detection of glucose in real saliva. Also, the individual SG levels at fasting are found to be unique and relatively consistent over time if subjects’ health conditions are unchanged. This discovery reveals that fasting SG levels are a crucial indicator of health conditions and can potentially be used for diabetes management. In the near future, it will be important to extend clinical trials that include diabetics to verify: (1) the efficiency of using SG levels to predict BG levels in real time; (2) whether sensors can detect extremely low/high SG levels as indications of dangerous hypoglycemia/hyperglycemia conditions. In conclusion, noninvasive SG glucose sensors have successfully been applied to measure a person’s glucose level. The two-minute saliva test, including the preparation of samples, has great potential to be a viable alternative to BG measurement at fasting, and can even be applied for national screening programs for undiagnosed diabetes.

## Figures and Tables

**Table 1:** Overview of screen-printed glucose biosensors.

Substrate	Electrode	Immobilization Method	Detection Range (mg/dL)	Reponse Time (Second)	Specimen	Generation	Reference
PET	RuO_2_	GOx /Nafion	100–400	100	buffer	1st	[18]
PPy	Pt	PPD/GOx	9–540	N/A	buffer	1st	[19]
Paper	Graphite	ferricyanide/GOx	up to 500	60	buffer	2nd	[20]
					blood		
PC	Graphite	HRP/PEGDGE/GOx	9–540	N/A	buffer	2nd	[21]
		/glutaraldehyde/BSA					
PET	Carbon	pyocyanin/GOx	18–360	120	buffer	2nd	[22]
					soft drinks		
Ceramic	Carbon	Tin(IV) oxide/GOx/Nafion	up to 200	N/A	blood	2nd	[23]
Unknown	Carbon	Rucoplex	100–800	2	blood	2nd	[24]
		/Ru(NH_3_)_6_Cl_3_ or K_3_Fe(CN)_6_					
		/FADGDH/GOx					
Ceramic	Carbon	Rhodium dioxide/				2nd	[25]
		*m-phenylenediamine/GOx*	10–500	25	honey		
		*GDH/GOx/Nafion*	10–200	120			
		*glutaraldehyde/BSA/GOx*	10–200	30	syrup		
		*cellulose acetate/Nafion/GOx*	10–200	240			
		*pyrrole/GOx*	50–250	35			
Unknown	SEC	CTC/GOx/gel	18–720	N/A	buffer	3rd	[26]
Ceramic	Pt	PPy-polystyrensulfonate	up to 180	11	serum	3rd	[27]
		/PA/GOx					
Aluminum	Carbon	CNT/GOx/Nafion	1.8–16.2	20	buffer	3rd	[28]
**Ceramic**	**Pt**	**SWNT/(chitosan/gold**	**0.5–20**	**30**	**buffer**	**3rd**	**Current study**
		**nanoparticles/GOx)_3_**	**1.1–45**		**saliva**		

Abbreviations of Table 1: N/A, not applicable because glucose is measured from continuous flow; SWNT, single-walled carbon nanotubes; PET, polyethylene terephthalate; PC, polycarbonate; PPy, polypyrrole; PPD, poly(o-phenylenediamine); HRP, horseradish peroxidase; PEGDGE, polyethylene glycol diglycidyl ether; BSA, bovine serum albumin; Ru(NH_3_)_6_Cl_3_, hexaammineruthenium (III) chloride; K_3_Fe(CN)_6_, potassium ferricyanide; FAD, flavin adenine dinucleotide; FADGDH, FAD-dependent glucose dehydrogenase; GDH, glucose dehydrogenase; SEC, shapable electroconductive film, a polyanion-doped polypyrrole film; CTC, stable charge transfer complex, tetrathiafulvalene-tet-racyanoquinodimethane; PA, polyacrylamide; RuO_2_, ruthenium dioxide; GOx, glucose oxidase; Pt, platinum; CNT, carbon nanotubes.

**Table 2:** A comparison between sensors immobilized with and without single-walled carbon nanotubes.

Amperometric Test	Pt/SWNT/(CS/GNp/GOx)_3_	Pt/(CS/GNp/GOx)_3_
Detection range (glucose in PBS)	0–20 mg/dL	0–20 mg/dL
^a^ Repeatability	<10%	<20%
^b^ Limit of detection (LOD)	0.41 mg/dL	4.94 mg/dL
^c^ linearity	0.98	0.78

^a^ Repeatability was presented by the coefficient of variation produced from at least four single-use sensors; ^b^ LOD was calculated by dividing the sum of an average blank signal (*x*) plus three times the standard deviation of *x* by the slope of regression line; ^c^ The linearity was evaluated by the R-squared (R^2^), which was a statistical measure of how close the data were to the fitted regression line. SWNT, single-walled carbon nanotubes; PBS, phosphate buffered saline; Pt, platinum; CS, chitosan; GNp, gold nanoparticles; GOx, glucose oxidase.

**Table 3:** Ten healthy subjects’ blood glucose (BG)/salivary glucose (SG) ratios at fasting.

Subjects	Gender	Age	BG (mg/dL)	SG (mg/dL)	BG/SG Ratio
A	female	20s	92.7	1.43	64.8
B	female	20s	85.2	1.22	69.8
C	male	40s	96.5	1.39	69.6
D	male	50s	114.6	1.99	57.6
E	male	50s	108	2.4	45
F	male	50s	101	1.46	69.2
G	male	50s	99.7	1.43	69.7
H	male	20s	91.6	1.55	59.1
I	male	20s	100.3	1.73	58
J	female	20s	91.6	1.05	87.2

## References

[1] (2014). National Diabetes Statistics Report. http://www.cdc.gov/diabetes/pubs/statsreport14/national-diabetes-report-web.pdf.

[2] Kaufman E., Lamster I.B. (2002). The diagnostic application of saliva—A review. Crit. Rev. Oral Biol. Med..

[3] Agrawal R.P., Sharma N., Rathore M.S., Gupta V.B., Jain S., Agarwal V., Goyal S. (2013). Noninvasive method for glucose level estimation by saliva. J. Diabetes Metab..

[4] Mirzaii-Dizgah I., Mirzaii-Dizgah M.R., Mirzaii-Dizgah M.H. (2013). Stimulated saliva glucose as a diagnostic specimen for diabetes mellitus. J. Arch. Mil. Med..

[5] Mitsumori M., Yamaguchi M., Kano Y. A new approach to noninvasive measurement of blood glucose using saliva analyzing systems. Proceedings of the 20th Annual International Conference of the Engineering in Medicine and Biology Society.

[6] Yamaguchi M., Mitsumori M., Kano Y. Development of noninvasive procedure for monitoring blood glucose levels using saliva. Proceedings of the 20th Annual International Conference of the Engineering in Medicine and Biology Society.

[7] Yeh C.K., Christodoulides N.J., Floriano P.N., Miller C.S., Ebersole J.L., Weigum S.E., Floriano P.N., Miller C.S., Ebersole J.L., Weigum S.E. (2010). Current development of saliva/oral fluid-based diagnostics. Tex. Dent. J..

[8] Zeller H., Novak P., Landgraf R. (1989). Blood glucose measurement by infrared spectroscopy. Int. J. Artif. Organs.

[9] D’Auria S., Lakowicz J.R. (2001). Enzyme fluorescence as a sensing tool: New perspectives in biotechnology. Curr. Opin. Biotechnol..

[10] Shao J., Lin M., Li Y., Li X., Liu J., Liang J., Yao H. (2012). *In vivo* blood glucose quantification using raman spectroscopy. PLoS ONE.

[11] Mclntosh T.S., Davis H.M., Matthews D.E. (2002). A liquid chromatography-mass spectrometry method to measure stable isotopic tracer enrichments of glycerol and glucose in human serum. Anal. Biochem..

[12] Vahjudi P.N., Patterson M.E., Lim S., Yee J.K., Mao C.S., Lee W.N. (2010). Measurement of glucose and fructose in clinical samples using gas chromatography/mass spectrometry. Clin. Biochem..

[13] Wang J., Zhang X., Ju H., Wang J. (2008). Electrochemical Glucose Biosensors. Sensors, Biosensors and Their Biomedical Applications.

[14] Lubrano G.J., Guilbault G.G. (1978). Glucose and l-amino acid electrodes based on enzyme membranes. Anal. Chim. Acta.

[15] Yoo E.H., Lee S.Y. (2010). Glucose biosensors: An overview of use in clinical practice. Sensors.

[16] Ghindilis L.A., Atanasov P., Wilkins E. (1997). Enzyme-catalyzed direct electron transfer: Fundamentals and analytical applications. Electroanalysis.

[17] De Almeida Pdel V., Grégio A.M., Machado M.A., de Lima A.A., Azevedo L.R. (2008). Saliva composition and functions: A comprehensive review. J. Contemp. Dent. Pract..

[18] Chou J., Tsai Y., Cheng T., Liao Y., Ye G., Yang S. (2014). Fabrication of arrayed flexible screen-printed glucose biosensor based on microfluidic framework. IEEE Sens. J..

[19] Jing J., Hong Y. (2000). Amperometric glucose sensor based on coimmobilization of glucose oxidase and poly(p-phenylenediamine) at a platinum microdisk electrode. Anal. Biochem..

[20] Nie Z., Deiss F., Liu X., Akbulut O., Whitesides G.M. (2010). Integration of paper-based microfluidic devices with commercial electrochemical readers. Lab Chip.

[21] Liu J., Sun S., Liu C., Wei S. (2011). An amperometric glucose biosensor based on a screen-printed electrode and os-complex mediator for flow injection analysis. Measurement.

[22] Ohfuji K., Sato N., Hamada-sato N., Kobayashi T., Imada C., Okuma H., Watanabe E. (2004). Construction of a glucose sensor based on a screen-printed electrode and a novel mediator pyocyanin from pseudomonas aeruginosa. Biosens. Bioelectron..

[23] Berisha L., Kalcher K., Hajrizi A., Arbneshi T. (2013). A new biosensor for glucose based on screen-printed carbon electrodes modified with Tin (IV)-oxide. Am. J. Anal. Chem..

[24] Yamaoka H., Sode K. (2007). A disposable electrochemical glucose sensor using catalytic subunit of novel thermostable glucose dehydrogenase. Open Biotechnol. J..

[25] Polan V., Soukup J., Vytřas K. (2010). Screen-printed carbon electrodes modified by rhodium dioxide and glucose dehydrogenase. Enzyme Res..

[26] Khan G.F., Ohwa M., Wernet W. (1996). Design of a stable charge transfer complex electrode for a third-generation amperometric glucose sensor. Anal. Chem..

[27] Retama J.R., Cabarcos E.L., Mecerreyes D., López-ruiz B. (2004). Design of an amperometric biosensor using polypyrrole-microgel composites containing glucose oxidase. Biosens. Bioelectron..

[28] Yang T., Hung C., Ke J., Zen J. (2008). An electrochemically preanodized screen-printed carbon electrode for achieving direct electron transfer to glucose oxidase. Electrochem. Commun..

[29] (2014). Glucose Colorimetric/Fluorometric Assay Kit. http://www.biovision.com/manuals/K606.pdf.

[30] Du Y., Zhang W., Wang M.L. (2016). An on-chip disposable salivary glucose sensor for diabetes control. J. Diabetes Sci. Technol..

[31] (2016). Whatman^®^ Westran^®^ PVDF Membranes. http://www.sigmaaldrich.com/catalog/product/aldrich/z671010?lang=en®ion=US.

[32] Costa R.R., Mano J.F., Gaharwar A.K., Sant S., Hancock M.J., Hacking S.A. (2013). Layer-by-layer self-assembly techniques for nanostructured devices in tissue engineering. Nanomaterials in Tissue Engineering: Characterization, Fabrication and Applications.

[33] Kumara M.T., Tripp B.C., Muralidharan S. (2007). Layer-by-layer assembly of bioengineered flagella protein nanotubes. Biomacromolecules.

[34] Rubner M.F., Cohen R.E., Decher G., Schlenoff J.B. (2012). Layer-by-layer processed multilayers: Challenges and opportunities. Multilayer Thin Films: Sequential Assembly of Nanocomposite Materials.

[35] Wang Y., Yao Y. (2012). Direct electron transfer of glucose oxidase promoted by carbon nanotubes is without value in certain mediator-free applications. Microchim. Acta.

[36] Andzane J., Tobin J.M., Li Z., Prikulis J., Baxendale M., Olin H., Holmes J.D., Erts D. (2007). Selection of application specific single and multi walled carbon nanotubes by *in situ* characterization of conductive and field emission properties. AZojono-J. Nanotechnol. Online.

[37] Tang J.L., Wang J.N. (2008). Chemical sensing sensitivity of long-period grating sensor enhanced by colloidal gold nanoparticles. Sensors.

[38] Zhang W., Wang M.L. (2014). Saliva Glucose Monitoring System. U.S. Patent.

[39] Zhang W., Du Y., Wang M.L. (2015). Noninvasive glucose monitoring using saliva nano-biosensor. Sens. Biosens. Res..

[40] Zhang W., Du Y., Wang M.L. (2015). On-chip ultra-sensitive glucose sensing using multilayer films composed of single-walled carbon nanotubes-gold nanoparticles-and glucose oxidase. Sens. Biosens. Res..

[41] Petkova G.A., Záruba C.K., Zvátora P., Král V. (2012). Gold and silver nanoparticles for biomolecule immobilization and enzymatic analysis. Nanoscale Res. Lett..

[42] (2002). Product Information. http://www.sigmaaldrich.com/content/dam/sigma-aldrich/docs/Sigma/Product_Information_Sheet/2/g6125pis.pdf.

[43] Ma S., Mu J., Qu Y., Jiang L. (2009). Effect of refluxed sliver nanoparticles on inhibition and enhancement of enzymatic activity of glucose oxidase. Colloids Surf. A Physicochem. Eng. Asp..

[44] Ruzgas T., Csöregi E., Emnéus J., Gorton L., Marko-Varga G. (1996). Peroxidase-modified electrodes: Fundamentals and application. Anal. Chim. Acta.

[45] Paloma Y., Jordi R., Jose M.P., Xavier R. (2010). Electrochemical sensing based on carbon nanotubes. Trends Anal. Chem..

[46] Dai M., Maxwell S., Vogt B.D., La-Belle J.T. (2012). Mesoporous carbon amperometric glucose sensors using inexpensive, commercial methacrylate-based binders. Anal. Chim. Acta.

[47] (2014). Diagnosing Diabetes and Learning about Prediabetes. http://www.diabetes.org/diabetes-basics/diagnosis/.

[48] Bower B.F., Moore R.E., McClatchey K.D. (2002). The interpretation of laboratory tests 97 György Abel and Michael Laposata. Clinical Laboratory Medicine.

